# Two Novel Mutations in the *EYS* Gene Are Possible Major Causes of Autosomal Recessive Retinitis Pigmentosa in the Japanese Population

**DOI:** 10.1371/journal.pone.0031036

**Published:** 2012-02-17

**Authors:** Katsuhiro Hosono, Chie Ishigami, Masayo Takahashi, Dong Ho Park, Yasuhiko Hirami, Hiroshi Nakanishi, Shinji Ueno, Tadashi Yokoi, Akiko Hikoya, Taichi Fujita, Yang Zhao, Sachiko Nishina, Jae Pil Shin, In Taek Kim, Shuichi Yamamoto, Noriyuki Azuma, Hiroko Terasaki, Miho Sato, Mineo Kondo, Shinsei Minoshima, Yoshihiro Hotta

**Affiliations:** 1 Department of Ophthalmology, Hamamatsu University School of Medicine, Hamamatsu, Japan; 2 Laboratory for Retinal Regeneration, RIKEN Center for Developmental Biology, Kobe, Japan; 3 Department of Ophthalmology, Kyungpook National University Hospital, Daegu, Korea; 4 Department of Ophthalmology, Institute of Biomedical Research and Innovation Hospital, Kobe, Japan; 5 Department of Otolaryngology, Hamamatsu University School of Medicine, Hamamatsu, Japan; 6 Department of Ophthalmology, Nagoya University Graduate School of Medicine, Nagoya, Japan; 7 Department of Ophthalmology and Laboratory of Cell Biology, National Center for Child Health and Development, Tokyo, Japan; 8 Department of Photomedical Genomics, Basic Medical Photonics Laboratory, Medical Photonics Research Center, Hamamatsu University School of Medicine, Hamamatsu, Japan; 9 Department of Ophthalmology and Visual Science, Chiba University Graduate School of Medicine, Chiba, Japan; Emory University School Of Medicine, United States of America

## Abstract

Retinitis pigmentosa (RP) is a highly heterogeneous genetic disease including autosomal recessive (ar), autosomal dominant (ad), and X-linked inheritance. Recently, arRP has been associated with mutations in *EYS* (Eyes shut homolog), which is a major causative gene for this disease. This study was conducted to determine the spectrum and frequency of *EYS* mutations in 100 Japanese arRP patients. To determine the prevalence of *EYS* mutations, all *EYS* exons were screened for mutations by polymerase chain reaction amplification, and sequence analysis was performed. We detected 67 sequence alterations in *EYS*, of which 21 were novel. Of these, 7 were very likely pathogenic mutations, 6 were possible pathogenic mutations, and 54 were predicted non-pathogenic sequence alterations. The minimum observed prevalence of distinct *EYS* mutations in our study was 18% (18/100, comprising 9 patients with 2 very likely pathogenic mutations and the remaining 9 with only one such mutation). Among these mutations, 2 novel truncating mutations, c.4957_4958insA (p.S1653KfsX2) and c.8868C>A (p.Y2956X), were identified in 16 patients and accounted for 57.1% (20/35 alleles) of the mutated alleles. Although these 2 truncating mutations were not detected in Japanese patients with adRP or Leber's congenital amaurosis, we detected them in Korean arRP patients. Similar to Japanese arRP results, the c.4957_4958insA mutation was more frequently detected than the c.8868C>A mutation. The 18% estimated prevalence of very likely pathogenic mutations in our study suggests a major involvement of *EYS* in the pathogenesis of arRP in the Japanese population. Mutation spectrum of *EYS* in 100 Japanese patients, including 13 distinct very likely and possible pathogenic mutations, was largely different from the previously reported spectrum in patients from non-Asian populations. Screening for c.4957_4958insA and c.8868C>A mutations in the *EYS* gene may therefore be very effective for the genetic testing and counseling of RP patients in Japan.

## Introduction

Retinitis pigmentosa (RP [MIM 268000]) is a highly heterogeneous genetic disease characterized by night blindness and visual field constriction leading to severe visual impairment. The disease appears with different modes of inheritance including autosomal recessive (ar), autosomal dominant (ad), and X-linked, and currently over half of cases are isolated in Japan.

To date, 53 causative genes and 7 loci of RP have been identified (http://www.sph.uth.tmc.edu/Retnet/), including the eyes shut homolog (*EYS*) gene encoding an ortholog of *Drosophila* spacemaker (spam), a protein essential for photoreceptor morphology. *EYS* spans over 2 Mb, making it one of the largest known genes expressed in the human eye [1], [2]. *EYS* gene mutations, primarily truncating and some missense mutations, have been detected in arRP families of different ancestral origin and have reported to account for 5–16% of arRP [3]–[6]. Most gene mutations (e.g., *RHO*, *PRPH2*, *PRPF31*, *RP1*, and *IMPDH1*) have been found in Japanese patients with adRP, with few reports describing mutations in arRP [7], [8]. Therefore, the genes causing arRP in most Japanese families have yet to be identified.

In this study, we screened all *EYS* gene exons in 100 unrelated Japanese RP patients. We found 2 novel truncating *EYS* gene mutations that were surprisingly related to 16% of Japanese arRP patients, but were not detected in Japanese patients with either adRP or Leber's congenital amaurosis (LCA [MIM204000], the earliest onset and most severe form of hereditary retinal dystrophy with several clinical features overlapping with those of RP). Additionally, these mutations were also detected in 9% of Korean arRP patients.

## Methods

### Patients and clinical evaluation

We screened all *EYS* gene exons in 100 unrelated Japanese RP patients with no systemic manifestations, excluding families with obvious autosomal dominant inheritance. Some pedigrees showed a pattern compatible with the recessive mode of inheritance; the other patients were considered isolated cases. In addition, 200 unrelated and non-RP Japanese individuals were screened as controls to evaluate the frequency of the mutations found in the patient samples. We also screened a part of *EYS* gene exons 26 and 44 in 19 unrelated Japanese adRP patients, 28 unrelated Japanese LCA patients, and 32 unrelated Korean arRP patients. The 19 Japanese adRP patients had already been screened for some principal adRP-causing genes, but the pathogenic mutations have not yet been detected. Examples of the screening list for adRP-causing genes and targeted exons include exon 3 and 4 in *RP1*; exon 1, 2, 3, 4, and 5 in *RHO*; exon 1, 2, and 3 in *PRPH2*; exon 2, 3, and 4 in *CRX*; exon 11 in *PRPF3*; exon 10, 11, and 12 in *IMPDH1*; exon 2 in *NRL*; exon 43 in *PRPF8*; exon 1 and 2 in *ROM1*; exon 5 and 6 in *RP9*; exon 2, 3, 5, 6, 7, 8, 11, and 12 in *PRPF31*; exon 11 and 15 in *SEMA4A*; exon 1 in *CA4*; exon 3 in *GUCA1B*; exon 3 in *SP4*; and exon 3 in *TOPORS*.

Japanese RP patients were examined either at the Department of Ophthalmology, Hamamatsu University Hospital in Hamamatsu (by YH), Department of Ophthalmology, Kobe City Medical Center General Hospital in Kobe (by MT), or Department of Ophthalmology, Nagoya University Hospital in Nagoya (by MK). Patients' origin varied widely, from the Tokyo to Osaka areas in Japan. Japanese LCA patients were examined at the Department of Ophthalmology and Laboratory of Cell Biology, National Center for Child Health and Development in Tokyo (by NA). LCA patients' origin varied widely, from all over Japan except the Okinawa islands. Meanwhile, Korean RP patients were examined at the Department of Ophthalmology, Kyungpook National University Hospital in Daegu (by ITK). The Korean patients' origin varied widely, from Daegu to Yeongju and Pohang areas in Gyeongsangbuk-do, Korea. A full ophthalmic examination was performed. Clinical diagnosis for RP was based on visual field, fundus examination, and electroretinogram findings, and clinical diagnosis for LCA was based on fundus examination and electroretinogram findings.

### Ethics statements

This study was approved by the Institutional Review Board for Human Genetic and Genome Research at the 6 participating institutions (Hamamatsu University School of Medicine, RIKEN Center for Developmental Biology, Nagoya University Graduate School of Medicine, National Center for Child Health and Development, Chiba University Graduate School of Medicine, and Kyungpook National University Hospital), and its procedures conformed to the tenets of the Declaration of Helsinki. Written informed consent was obtained from all participants before molecular genetic studies.

### Mutation analysis

Genomic DNA in Japanese samples was extracted from the peripheral lymphocytes using standard procedures. In Korean samples, whole blood samples were collected on FTA cards (GE Healthcare). Blood samples were spotted onto the cards and air-dried for 1 h at room temperature. For polymerase chain reaction (PCR) amplification, a 1.2-mm disk was punched from a dried blood spot using a Harris micro-punch tool (GE Healthcare) and processed according to the manufacturer's instructions. PCR was performed using the KOD -Plus- ver. 2 PCR kit (Toyobo) with the primer sets described in Table S1 for 35 cycles of 98°C for 10 s, 60°C for 30 s, and 68°C for 1 min in an automated thermal cycler (GeneAmp PCR System 9700; Applied Biosystems). PCR products were purified with Wizard SV Gel and PCR Clean-up System (Promega) or treated with Exonuclease I and Antarctic Phosphatase (New England Biolabs). Direct sequencing was performed using the BigDye Terminator v3.1 Cycle Sequencing Kit on an ABI3100 autosequencer (Applied Biosystems). For Japanese arRP patients, all 44 exons, including 3 non-coding exons (exons 1–3) that cover the 5′ untranslated region and 41 coding exons (exons 4–44), were analyzed in both sense and antisense directions. For Japanese adRP and LCA patients, and Korean arRP patients, parts of exons 26 and 44 were analyzed (Table S1).

### Assessment of pathogenicity

A sequence variant was considered pathogenic if it represented a truncating mutation (nonsense or frameshift), large-scale deletion mutation, or missense mutation affecting a conserved amino acid residue and did not appear in control samples (number of alleles studied ≤400) and/or in a public SNP database (http://www.ncbi.nlm.nih.gov/projects/SNP/). Particularly, missense mutations were considered pathogenic if found together with a second variant, especially if it was truncating. As reference data, we employed 4 computational algorithms to evaluate the pathogenicity of missense mutations: SIFT (http://sift.jcvi.org/www/SIFT_seq_submit2.html), PolyPhen2 (http://genetics.bwh.harvard.edu/pph2/), PMut (http://mmb.pcb.ub.es/PMut/), and SNAP (http://rostlab.org/services/snap/).

## Results

### Mutation analysis

**Figure 1:**
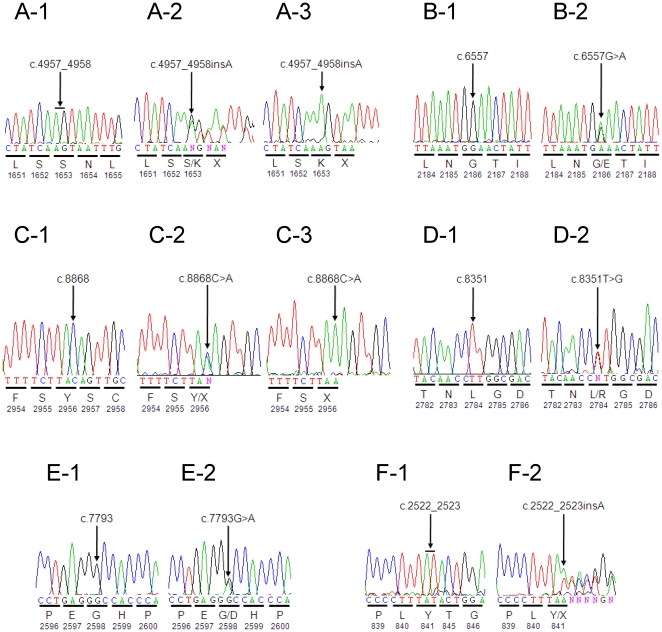
Electropherograms of the 6 likely pathogenic *EYS* mutations. Partial sequence of the *EYS* gene showing the normal control sequences (A-1 through F-1), heterozygous mutation sequences (A-2 through F-2), and homozygous mutation sequences (A-3 and C-3). Deduced amino acids are indicated under the sequence trace. The mutation location is indicated either by an arrow (for a nucleotide change) or a horizontal line (to show 2 nucleotides between which the insertion occurred). (A) c.4957_4958insA; p.S1653KfsX2 (Exon 26), (B) c.6557G>A; p.G2186E (Exon 32), (C) c.8868C>A; p.Y2956X (Exon 44), (D) c.8351T>G; p.L2784R (Exon 44), (E) c.7793G>A; p.G2598D (Exon 40), (F) c.2522_2523insA; p.Y841X (Exon 16).

Mutation analysis of *EYS* in 100 unrelated Japanese patients revealed 7 very likely pathogenic mutations in 18 patients (18%). Of these 18 patients, a second mutant allele could not be detected in 9 patients. The very likely pathogenic mutations consisted of 3 truncating mutations, 1 deletion mutation, 2 missense mutations, and 1 previously described mutation (Fig. 1, Table 1, and Table 2). In addition, we also identified 6 possible pathogenic mutations in 8 separate patients (Table 1 and Table 2).

**Table 1:** Mutation spectrum of the *EYS* gene in Japanese families.

Family ID	Nucleotide change	Predicted effect	Domain^a^	Location in gene	Type of change	Reference
Families with very likely pathogenic mutations and both alleles affected						
RP3H^b^	c.4957_4958insA/c.4957_4958insA	p.S1653KfsX2/p.S1653KfsX2	Close to coiled-coil/Close to coiled-coil	Exon 26/Exon 26	Homozygous	This study
RP48K^b^	c.4957_4958insA/c.4957_4958insA	p.S1653KfsX2/p.S1653KfsX2	Close to coiled-coil/Close to coiled-coil	Exon 26/Exon 26	Homozygous	This study
RP54K	c.4957_4958insA/c.4957_4958insA	p.S1653KfsX2/p.S1653KfsX2	Close to coiled-coil/Close to coiled-coil	Exon 26/Exon 26	Homozygous	This study
RP44K	c.4957_4958insA/c.6557G>A	p.S1653KfsX2/p.G2186E	Close to coiled-coil/Laminin G	Exon 26/Exon 32	Heterozygous/Heterozygous	This study/Abd El-Aziz et al., 2010; Littink et al., 2010; This study
RP56K^b^	c.4957_4958insA/c.8351T>G	p.S1653KfsX2/p.L2784R	Close to coiled-coil/Laminin G	Exon 26/Exon 44	Compound Heterozygous	This study
RP87N	c.4957_4958insA/c.7793G>A	p.S1653KfsX2/p.G2598D	Close to coiled-coil/Close to Laminin G	Exon 26/Exon 40	Heterozygous/Heterozygous	This study
RP81K^b^	c.2522_2523insA/c.6557G>A	p.Y841X/p.G2186E	EGF/Laminin G	Exon 16/Exon 32	Compound Heterozygous	This study/Abd El-Aziz et al., 2010; Littink et al., 2010; This study
RP21H	deletion exon32/deletion exon32	p.D2142_S2191delinsG/p.D2142_S2191delinsG	Laminin G/Laminin G	Exon 32/Exon 32	Homozygous	This study
RP35K	c.8868C>A/c.8868C>A	p.Y2956X/p.Y2956X	EGF/EGF	Exon 44/Exon 44	Homozygous	This study
Families with single very likely pathogenic mutations						
RP1H	c.4957_4958insA	p.S1653KfsX2	Close to coiled-coil	Exon 26	Heterozygous	This study
RP6H	c.4957_4958insA	p.S1653KfsX2	Close to coiled-coil	Exon 26	Heterozygous	This study
RP12H	c.4957_4958insA	p.S1653KfsX2	Close to coiled-coil	Exon 26	Heterozygous	This study
RP51K	c.4957_4958insA	p.S1653KfsX2	Close to coiled-coil	Exon 26	Heterozygous	This study
RP96H	c.4957_4958insA	p.S1653KfsX2	Close to coiled-coil	Exon 26	Heterozygous	This study
RP100N	c.4957_4958insA	p.S1653KfsX2	Close to coiled-coil	Exon 26	Heterozygous	This study
RP8H	c.8868C>A	p.Y2956X	EGF	Exon 44	Heterozygous	This study
RP25H	c.8868C>A	p.Y2956X	EGF	Exon 44	Heterozygous	This study
RP80K^b^	c.8868C>A	p.Y2956X	EGF	Exon 44	Heterozygous	This study
Families with single possible pathogenic mutations						
RP4H	c.9272T>C	p.I3091T	Laminin G	Exon 44	Heterozygous	This study
RP9H	c.8875C>A	p.L2959M	EGF	Exon 44	Heterozygous	This study
RP49K	c.9272T>C	p.I3091T	Laminin G	Exon 44	Heterozygous	This study
RP53K	c.5884A>G	p.T1962A	Laminin G	Exon 28	Heterozygous	This study
RP55K	c.9272T>C	p.I3091T	Laminin G	Exon 44	Heterozygous	This study
RP74K	c.5404C>T	p.L1802F	Close to Laminin G	Exon 26	Heterozygous	This study
RP79K	c.77G>A	p.R26Q	Close to signal peptide cleavage site	Exon 4	Heterozygous	This study
RP83K	c.2923T>C	p.C975R	EGF	Exon 19	Heterozygous	This study

**Figure 2:**
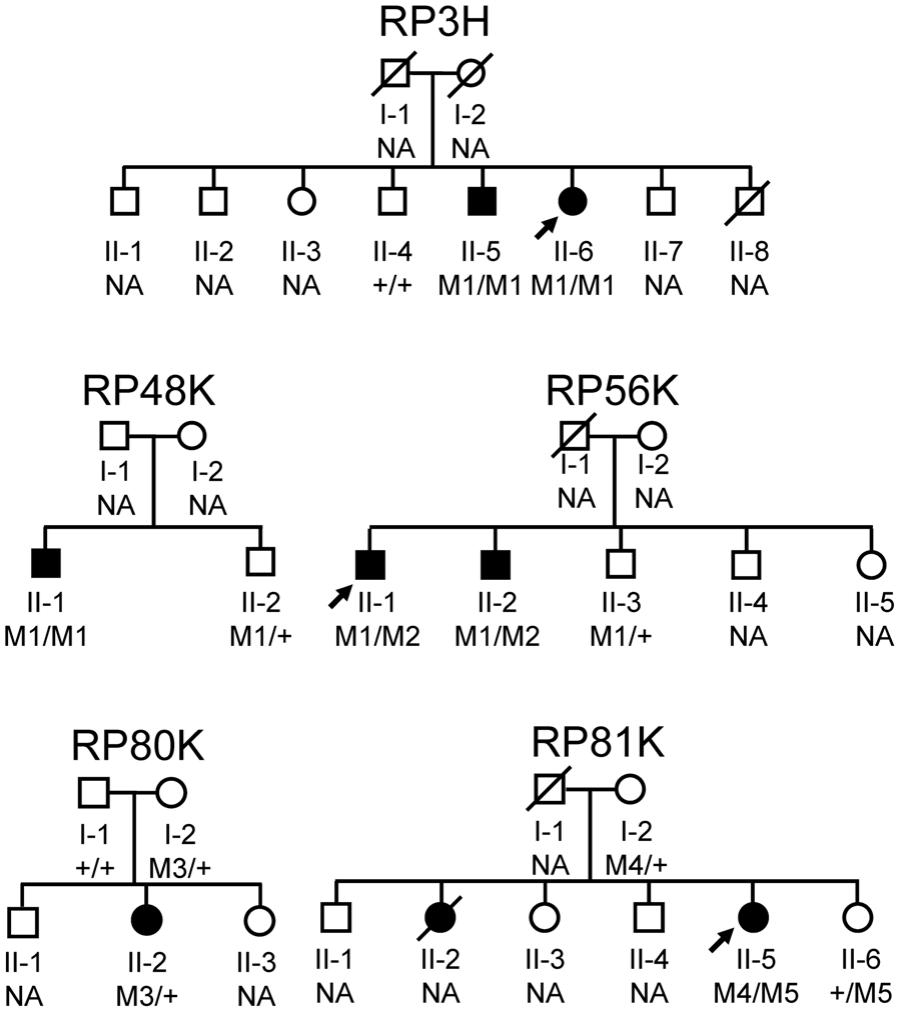
Pedigrees of the families that was available for mutation analysis. Below the individuals, genotypes are presented for either p.S1653KfsX2 (M1), p.L2784R (M2), p.Y2956X (M3), p.Y841X (M4), or p.G2186E (M5) detected to segregate with RP. M1/M1 represents homozygous mutation. M1/+ indicates heterozygous carriers, +/+ indicates individuals carrying 2 wild-type alleles, whereas M1/M2 represents individuals presenting both mutations as compound heterozygous. Square boxes indicate men, circles denote women, and affected individuals are pointed out by a black symbol. Slashed symbols indicate deceased individuals. The probands are indicated with an arrow. NA denotes unavailable DNA samples.

**Figure 3:**
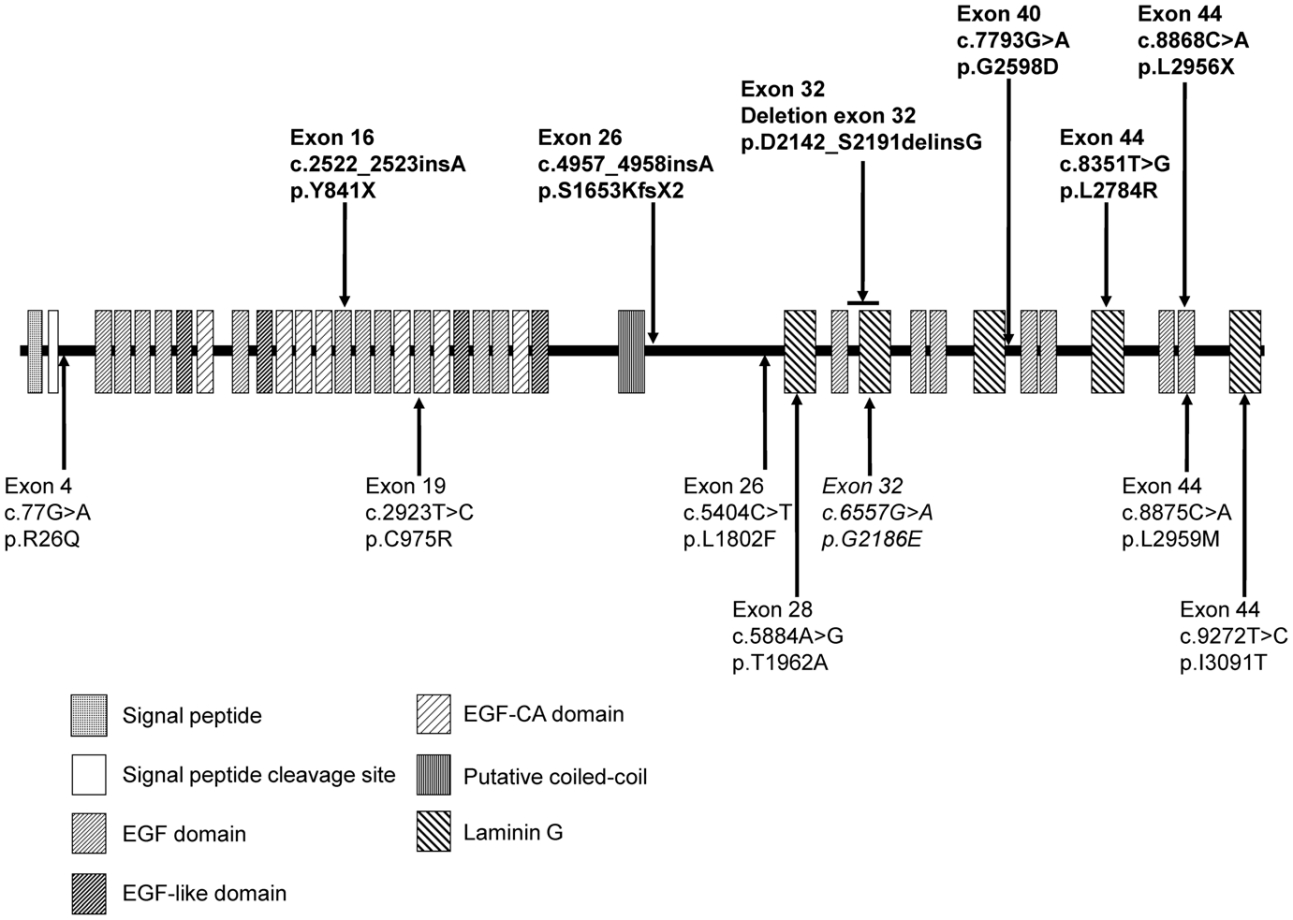
Predicted domain structure and distribution of identified *EYS* mutations. SMART (http://smart.embl-heidelberg.de/) and Pfam (http://pfam.sanger.ac.uk/) were used to search protein functional domains. A coiled-coil domain identified by Barragán et al. (2010) between the EGF-like domain and laminin G domain was also indicated. Novel very likely pathogenic mutations, novel possible pathogenic mutations, and a previously described mutation are shown in bold, normal, and italic type, respectively. Six out of 9 missense mutations were found in the EGF or laminin G domains. Furthermore, 7 were located in the latter half of the protein between the putative coiled-coil region and C-terminus.

Nucleotide numbering reflects cDNA numbering with +1 corresponding to the A of the ATG translation initiation codon in the reference sequence FM209056, according to the nomenclature recommended by the Human Genome Variation Society (www.hgvs.org/mutnomen). The initiation codon is codon 1. None of these 13 mutations were found in the Japanese controls.

a EYS has a signal peptide, a putative coiled-coil, 29 EGF, and 5 Laminin G domains. See Fig. 3.

b Segregation analysis has been performed. See Fig. 2.

In RP56K and RP81K, 2 pathogenic alleles were considered to be on different chromosomes (compound heterozygous). See Fig. 2.

**Table 2:** Summary of the very likely and possible pathogenic mutations identified in 100 Japanese arRP patients.

							Allele frequency					Computational prediction^c^			
		Nucleotide change	Predicted effect	Location in gene	Domain^a^	Conservation in hu/o/m/ho/d/op/p/c/z/dr^b^	Control	Patient	Family ID	Reference	Species	SIFT	PolyPhen2 (HumDiv)	PMut	SNAP
**Very likely pathogenic mutations**	**Insertion**	c.2522_2523insA	p.Y841X	Exon 16	EGF	not applicable	0/400	1/200	RP81K	This study	Japanese				
		c.4957_4958insA	p.S1653KfsX2	Exon 26	Close to coiled-coil	not applicable	0/400	15/200	RP1H, RP3H, RP6H, RP12H, RP48K, RP51K, RP54K, RP44K, RP56K, RP87N, RP96H, RP100N	This study	Japanese				
	**Nonsense**	c.8868C>A	p.Y2956X	Exon 44	EGF	not applicable	0/400	5/200	RP8H, RP25H, RP35K, RP80K	This study	Japanese				
	**Deletion**	Deletion exon 32	p.D2142_S2191delinsG	Exon 32	Laminin G	not applicable	0/200^d^	2/200	RP21H	This study	Japanese				
	**Missense**	c.6557G>A	p.G2186E	Exon 32	Laminin G	G/G/G/G/G/-/-/-/-/-	0/400	2/200	RP44K, RP81K	Abd El-Aziz et al., 2010; Littink et al., 2010; This study	Chinese, South Korean/American, Japanese		Probably damaging	Pathological	Non-neutral
		c.7793G>A	p.G2598D	Exon 40	Close to Laminin G	G/G/G/-/-/-/-/G/I/T	0/400	1/200	RP87N	This study	Japanese		Probably damaging		Non-neutral
		c.8351T>G	p.L2784R	Exon 44	Laminin G	L/L/L/L/L/L/L/L/L/G	0/400	1/200	RP56K	This study	Japanese		Probably damaging		Non-neutral
**Possible pathogenic mutations**	**Missense**	c.77G>A	p.R26Q	Exon 4	Close to signal peptide cleavage site	R/R/R/K/K/-/-/-/-/-	0/400	1/200	RP79K	This study	Japanese	Affected protein function		Pathological	
		c.2923T>C	p.C975R	Exon 19	EGF	C/C/C/-/-/-/-/-/-/-	0/400	1/200	RP83K	This study	Japanese		Possibly damaging	Pathological	Non-neutral
		c.5404C>T	p.L1802F	Exon 26	Close to Laminin G	L/L/L/-/-/-/-/-/-/-	0/400	1/200	RP74K	This study	Japanese		Possibly damaging		
		c.5884A>G	p.T1962A	Exon 28	Laminin G	T/T/T/T/-/-/-/-/-/-	0/400	1/200	RP53K	This study	Japanese		Possibly damaging		
		c.8875C>A	p.L2959M	Exon 44	EGF	L/L/L/L/L/L/A/V/-/S	0/400	1/200	RP9H	This study	Japanese		Possibly damaging		
		c.9272T>C	p.I3091T	Exon 44	Laminin G	I/I/I/I/I/I/I/I/I/L	0/400	3/200	RP4H, RP49K, RP55K	This study	Japanese	Affected protein function	Probably damaging		

a EYS contains a signal peptide, a putative coiled-coil, 29 EGF, and 5 laminin G domains. See Fig. 3.

b hu/o/m/ho/d/op/p/c/z/dr denotes Human/Orangutan/Marmoset/Horse/Dog/Opossum/Platypus/Chicken/Zebrafish/Drosophila EYS orthologs, respectively. The hyphen (-) indicates that genomic sequence of corresponding region in the species was reported to be unknown [5].

c SIFT, PolyPhen2 (only the HumDiv data are shown), PMut, and SNAP were used as reference data to evaluate the pathogenicity of the missense mutations. c.77G>A, c.2923T>C, c.7793G>A, c.8351T>G, and c.9272T>C were predicted to be pathogenic by a number of different computational prediction programs. In addition, the c.6557G>A mutation, which had been previously reported as disease causing, was classified as pathogenic by the PolyPhen2, PMut, and SNAP programs.

d Homozygous exon 32 deletion mutation was not detected in 200 controls.

A novel truncating insertion, c.4957_4958insA, was detected in 12 patients and accounted for 15 of the 35 mutated alleles detected (42.9%) (Table 1 and Table 2). Three patients were homozygous for the c.4957_4958insA mutation, and the other 9 patients were heterozygous. Of the latter, 3 patients showed the second mutation while 6 did not. This insertion creates a frameshift mutation that predicts a premature stop at codon 1654 (p.S1653KfsX2). A novel truncating nonsense mutation c.8868C>A (p.Y2956X) was identified in 4 patients and accounted for 5 of the 35 mutated alleles detected (14.3%). Thus, these 2 novel truncating mutations were identified in 16 separate patients, resulting in a very high frequency of the 2 mutations in Japanese arRP patients.

### Families with very likely pathogenic mutations and both alleles affected

Nine of the 18 patients bearing very likely pathogenic mutations appeared to have both alleles affected, suggesting that they received one mutated allele from each unaffected parent (Table 1 and Table 2). In 4 patients (RP3H, RP48K, RP56K, and RP81K), segregation analysis was performed, and the 2 pathogenic alleles were considered to be on different chromosomes (Fig. 2).

1. In RP3H, proband (II-6) was homozygous for c.4957_4958insA. The mutation co-segregated with the phenotype: the unaffected brother (II-4) demonstrated wild-type alleles, while the affected brother (II-5) was homozygous for the mutation.
2. In RP48K, proband (II-1) was homozygous for c.4957_4958insA. The unaffected brother (II-2) was heterozygous for the mutation.
3. In RP56K, proband (II-1) was compound heterozygous for c.4957_4958insA and missense mutation c.8351T>G (p.L2784R). The mutation co-segregated with the phenotype: the affected brother (II-2) also showed both mutations, while the unaffected brother (II-3) was heterozygous for c.4957_4958insA.
4. In RP81K, proband (II-5) was compound heterozygous for truncating insertion c.2522_2523insA (p.Y841X) and missense mutation c.6557G>A (p.G2186E). This insertion results in premature termination of the encoded protein at codon 841 (p.Y841X). Missense mutation c.6557G>A has been previously reported as disease causing in one Korean/American and one Chinese patient [3], [6]. The unaffected mother (I-2) was heterozygous for c.2522_2523insA, while the unaffected sister (II-6) was heterozygous for c.6557G>A.

For the other patients, segregation analysis could not be performed due to difficulties in collecting samples from the families of patients (Table 1). RP54K and RP35K were homozygous for truncating mutation c.4957_4958insA and c.8868C>A, respectively. RP21H was homozygous for deletion in exon 32, an in-frame deletion that results in the replacement of amino acids from D2142 to S2191 with G2142 (p.D2142_S2191delinsG) and disrupts the second laminin G domain (Fig. 3). RP44K and RP87N were heterozygous for truncating and missense mutations, c.4957_4958insA/c.6657G>A (p.G2186E) and c.4957_4958insA/c.7793T>G (p.G2598D), respectively. None of these 7 very likely pathogenic mutations were found in the Japanese controls.

### Families with single novel very likely pathogenic mutations

The rest of the patients comprising the group with very likely pathogenic mutations presented only single truncating mutations (Table 1 and Table 2). RP1H, RP6H, RP12H, RP51H, RP96H, and RP100N were heterozygous for c.4957_4958insA. RP8H, RP25H, and RP80K were heterozygous for c.8868C>A. Segregation analysis was performed in patient RP80K. The unaffected father (I-1) demonstrated wild-type alleles, and the unaffected mother (I-2) was heterozygous for the mutation (Fig. 2). In RP96H, we found very likely pathogenic missense mutation c.8923T>C (p.F2975L), which was not detected in any of the 400 control alleles. However, as c.8923T>C has been described as rs79036642 in the dbSNP database, it was assigned to the group of possible non-pathogenic sequence alterations (Table 3).

**Table 3:** Summary of the possible non-pathogenic sequence alterations in the *EYS* gene identified in this study.

Gene exon	Nucleotide change	Predicted effect	Conservation in hu/o/m/ho/d/op/p/c/z/dr^a^	Patient frequency	Control frequency	SNP ID	Reference
Exon 1	c.-500A>G			13/200		rs1490127	Abd El-Aziz et al., 2010
Exon 4	c.334G>A	p.V112I	V/I/I/I/I/I/-/-/-/E	1/200	0/192	rs112609906	
	c.359C>T	p.T120M	T/T/T/T/T/A/-/-/-/I	60/200		rs12193967	Audo et al., 2010; Abd El-Aziz et al., 2010
	c.525_527delGGA	p.176delE	E/E/E/E/E/A/-/-/-/G	1/200	1/192		This study
Intron 5	c.863-23_863-22insTT			53/200		rs34154043	Abd El-Aziz et al., 2010
	c.863-23_863-22insTTT			44/200			This study
Exon 6	c.1005G>T	p.E335D	E/E/D/-/-/-/-/-/-/-	3/200		rs80095433	
Exon 7	c.1146T>C	p.N382N	N/N/T/-/-/-/-/-/-/-	97/200		rs974110	Audo et al., 2010; Abd El-Aziz et al., 2010
Intron 8	c.1300-3C>T			117/200		rs1936439	Audo et al., 2010; Abd El-Aziz et al., 2010
Exon 9	c.1382G>A	p.C461Y	C/C/Y/-/-/-/-/-/-/-	8/200	4/192	rs76754818	Littink et al., 2010
Intron 9	c.1599+96A>C			200/200		rs1502963	Abd El-Aziz et al., 2010
Intron 10	c.1600-38G>A			12/200		rs1502965	Abd El-Aziz et al., 2010
Exon 11	c.1712A>G	p.Q571R	Q/Q/Q/-/-/-/-/-/-/-	26/200		rs61753610	Audo I et al., 2010
Exon 12	c.1809C>T	p.V603V	V/V/V/-/-/-/-/-/-/-	178/200		rs9345601	Audo et al., 2010; Abd El-Aziz et al., 2010
	c.1891G>A	p.G631S	G/S/E/C/C/-/-/-/-/-	178/200		rs9342464	Audo et al., 2010; Abd El-Aziz et al., 2010
	c.1922A>T	p.E641V	E/E/E/E/E/-/-/-/-/-	18/200		rs17411795	Audo et al., 2010; Abd El-Aziz et al., 2010
	c.1985G>T	p.R662M	R/R/R/S/S/-/-/-/-/-	8/200	3/96		This study
Intron 12	c.2023+6_2023+7insT			175/200		rs67504324	
	c.2024-14C>T			3/200		rs45628235	
Intron 15	c.2382-26C>G			106/200		rs9445437	
Exon 16	c.2490T>C	p.P830P	P/P/P/P/P/P/P/Q/P/-	2/200	1/392		This study
	c.2528G>A	p.G843E	G/G/G/G/G/G/G/G/A/G	16/200	9/192	rs74419361	
	c.2555T>C	p.L852P	L/P/P/-/S/P/S/P/-/E	106/200		rs9294631	Audo et al., 2010; Abd El-Aziz et al., 2010
Intron 18	c.2846+52_2846+53insTAAT			120/200		rs66504228	Abd El-Aziz et al., 2010
	c.2847-24C>T			178/200		rs7743515	
Exon 19	c.2980C>G	p.P994A	P/P/P/-/-/-/-/-/-/-	3/200	2/192		This study
Intron 22	c.3444-5C>T			69/200		rs9445051	Audo et al., 2010; Abd El-Aziz et al., 2010
Intron 23	c.3568+60delA			1/200			This study
Exon 25	c.3787A>G	p.I1263V	I/V/V/V/V/-/-/-/-/I	36/200		rs17404123	Audo et al., 2010; Abd El-Aziz et al., 2010
	c.3809T>G	p.V1270G	V/V/V/V/V/-/-/-/-/P	1/200	1/192		This study
Intron 25	c.3877+17_22delAGATA			36/200			Barragán I et al., 2010
Exon 26	c.3906C>T	p.H1302H	H/H/H/H/H/-/-/-/-/S	10/200		rs12663916	Audo et al., 2010; Abd El-Aziz et al., 2010
	c.3936A>G	p.T1312T	T/A/T/A/A/-/-/-/-/S	10/200		rs12662610	Audo et al., 2010; Abd El-Aziz et al., 2010
	c.3973C>G	p.Q1325E	Q/E/K/K/K/-/-/-/-/S	12/200		rs12663622	Audo et al., 2010; Abd El-Aziz et al., 2010
	c.4026C>T	p.S1342S	S/S/S/S/S/-/-/-/-/A	10/200		rs12663619	Audo et al., 2010; Abd El-Aziz et al., 2010
	c.4081A>G	p.I1361V	I/I/T/V/V/-/-/-/-/S	12/200		rs17403955	Audo et al., 2010; Abd El-Aziz et al., 2010
	c.4256T>C	p.L1419S	L/S/S/S/S/L/S/V/Q/V	137/200		rs624851	Audo et al., 2010; Abd El-Aziz et al., 2010
	c.4352T>C	p.I1451T	I/T/T/K/K/-/-/-/-/T	13/200		rs62415828	Audo et al., 2010; Abd El-Aziz et al., 2010
	c.4543C>T	p.R1515W	R/R/R/R/R/-/-/-/-/H	36/200		rs62415827	Audo et al., 2010; Abd El-Aziz et al., 2010
	c.4549A>G	p.S1517G	S/G/D/T/T/-/-/-/-/H	36/200		rs62415826	Audo et al., 2010; Abd El-Aziz et al., 2010
	c.4593G>A	p.E1531E	E/E/E/E/E/-/-/-/-/Q	36/200		rs62415825	Audo et al., 2010; Abd El-Aziz et al., 2010
	c.5244A>C	p.L1748F	L/L/L/L/L/-/-/-/-/F	8/200		rs57312007	Audo I et al., 2010; Littink et al., 2010
	c.5617C>G	p.L1873V	L/L/L/P/P/-/-/-/-/I	38/200		rs16895517	Audo I et al., 2010
Exon 27	c.5705A>T	p.N1902I	N/N/N/N/N/P/-/R/-/A	90/200		rs9353806	Audo et al., 2010; Abd El-Aziz et al., 2010
Intron 28	c.5928-35T>C			118/200		rs587278	Abd El-Aziz et al., 2010
Intron 29	c.6078+68A>G			81/200		rs36133910	Abd El-Aziz et al., 2010
	c.6079-4_6079-3delTC			87/200		rs35395170	Audo I et al., 2010
Intron 34	c.6834+61T>G			60/200		rs66502009	Abd El-Aziz et al., 2010
Exon 35	c.6977G>A	p.R2326Q	R/R/R/L/L/L/L/L/I/L	95/200		rs4710457	Audo et al., 2010; Abd El-Aziz et al., 2010
Exon 37	c.7394C>G	p.T2465S	T/T/T/T/T/T/T/T/S/F	8/200	2/176		This study
Exon 39	c.7666A>T	p.S2556C	S/S/S/S/S/N/S/H/E/E	57/200		rs66462731	Audo et al., 2010; Abd El-Aziz et al., 2010; Barragán et al., 2010; Littink et al., 2010
Intron 41	c.8071+84T>G			53/200		rs4710257	Abd El-Aziz et al., 2010
Exon 44	c.8923T>C	p.F2975L	F/F/F/F/F/F/F/F/-/K	1/200	0/400	rs79036642	
	c.9300A>G	p.L3100L	L/L/L/L/L/L/L/L/V/I	4/200	2/192		This study

Fifty-four sequence alterations were identified in 100 patients. These alterations were predicted to be non-pathogenic for various reasons. Some have been reported as polymorphisms in previous reports. Newly identified alterations within the exons, except for c.334G>A and c.8923T>C, were also found in the control chromosome. The hyphen (-) indicates that genomic sequence of corresponding region in the species was reported to be unknown [5].

a hu/o/m/ho/d/op/p/c/z/dr denotes Human/Orangutan/Marmoset/Horse/Dog/Opossum/Platypus/Chicken/Zebrafish/Drosophila EYS orthologs, respectively.

### Families with single novel possible pathogenic mutations

A group of patients with possible pathogenic mutations had only single missense mutations (Table 1 and Table 2). We report 6 novel missense mutations in 8 different patients (Table 1 and Table 2), none of which were identified in the 400 Japanese control alleles. All amino acid residues affected by these mutations were compared with those encoded by orthologous genes of various vertebrates (orangutan, marmoset, horse, dog, opossum, platypus, chicken, and zebrafish) and *Drosophila* and found to be highly conserved across species (Table 2). The novel missense mutation c.2923T>C (p.C975R) was predicted to be pathogenic by 3 different computational prediction programs (PolyPhen2, PMut, and SNAP) (Table 2). RP4H, RP49K, and RP55K were heterozygous for the same missense mutation c.9272T>C (p.I3091T), which was predicted to be pathogenic by SIFT and PolyPhen2 programs (Table 2). In addition, 54 possible non-pathogenic sequence alterations were found, of which 9 were previously unreported (Table 3).

### Screening of the 2 truncating mutations

We focused on 2 truncating mutations, c.4957_4958insA in exon 26 and c.8868C>A in exon 44, which were identified in 16 separate Japanese arRP patients in this study. The frequency of the 2 mutations was very high in this Japanese arRP cohort. However, we did not detect the 2 mutations in 19 Japanese adRP patients and 28 LCA patients who were recruited and screened to evaluate the frequency of the mutations. We also recruited 32 unrelated Korean arRP patients and screened for the 2 *EYS* gene mutations. The c.4957_4958insA mutation was detected in 2 patients and accounted for 3 of 64 Korean patient alleles (4.7%). One patient was homozygous and the other was heterozygous. The c.8868C>A mutation was identified in 1 patient and accounted for 1 of the 64 Korean patient alleles (1.6%).

### Clinical findings

Nine Japanese patients with very likely pathogenic *EYS* gene mutations in both alleles, 9 Japanese patients with single very likely pathogenic changes, and a Korean patient with homozygous c.4957_4958insA mutation demonstrated classic RP with mostly night blindness as the initial symptom, followed by gradual constriction of the visual field. The fundus displayed bone spicules increasing in density with age and attenuated retinal vessels. Electroretinogram responses were not detectable, consistent with severe generalized rod-cone dysfunction. The remaining visual field determined using Goldmann kinetic perimetry with V-4 target ranged from approximately 10° to 60° of the central and inferior visual fields, respectively, in a 74-year-old woman (RP100N) to complete blindness in a 54-year-old man (RP21H). No remarkable clinical difference was observed between 9 patients with very likely pathogenic *EYS* gene mutations in both alleles and 9 patients with single very likely pathogenic changes.

## Discussion

This study is the first to analyze mutations in the *EYS* gene among Japanese arRP patients. We detected 67 sequence alterations in the *EYS* gene, of which 21 were novel. Of these, 7 were very likely pathogenic mutations, 6 were possibly pathogenic mutations, and 54 were possible non-pathogenic sequence alterations (Table 1, Table 2, and Table 3).

Considering only the very likely pathogenic mutations, the minimum observed prevalence of distinct *EYS* gene mutations in our study is 18% (18/100, 9 patients with 2 very likely pathogenic mutations and 9 with only one such mutations). Additionally, if the possible pathogenic mutations are included in the prevalence estimation, prevalence increases to 26% (26/100, with 17 of 26 patients presenting single mutations). The estimated prevalence in our study may be extremely high compared with those in the previous studies [3]–[6]. Until recently, mutations in 34 genes have been associated with arRP (http://www.sph.uth.tmc.edu/Retnet/). The most frequently mutated gene is *USH2A*, accounting for approximately 7% of arRP cases [9], [10], whereas most other genes contribute to only 1% to 2% of arRP cases [11]. The estimated prevalence of very likely and possible pathogenic mutations of the *EYS* gene in our study was 26%, suggesting its major involvement in the pathogenesis of arRP in the Japanese population.

We found that 16% of Japanese arRP patients displayed at least one c.4957_4958insA or c.8868C>A mutation, which accounted for 57.1% (15+5/35) of the mutated alleles. Thus, these mutations seem to be frequent among Japanese arRP patients. Previous studies employing Indonesian, Pakistani, Chinese, Israeli, Spanish, French, British, Dutch, and Palestinian RP patient populations have not detected them [3]–[6], [12]–[15]. Since the Japanese were divided into small semi-closed population groups among which intercommunication was quite less until the mid-20^th^ century, obvious or latent consanguineous marriages were carried out more frequently, leading to relatively high inbreeding levels in those populations. The frequency of the c.4957_4958insA and c.8868C>A mutations may result from a founder effect like that of the 2299delG *USH2A* gene mutation, which accounts for 44% of disease alleles in Danish and Norwegian patients with Usher syndrome type II [16].

We detected 13 different very likely and possible pathogenic mutations. Three were truncating mutations and accounted for 60% (21/35) of mutated alleles. Likewise, previous studies reported that most pathogenic mutations were truncated type (nonsense, deletion, insertion, or splicing) [3]–[6], [12]–[15]. Furthermore, c.6557G>A was the only mutation that was common between the Japanese and other populations. This mutation has been found in Korean/American and Chinese patients [3], [6]. These results indicate that the *EYS* gene mutation spectrum among Japanese patients largely differs from that among the previously mentioned non-Asian populations. The Japanese and Korean mutation spectrum may resemble each other, but an accurate comparison could not be made, because further *EYS* gene analysis of Korean RP patients is required to clarify this possibility.

A second mutant allele could not be detected by direct sequencing in 17 of 26 patients in our study. Previous studies reported 7 of 10 [3] and 9 of 17 [5] patients with heterozygous *EYS* gene mutation, implying that this finding could be due to relatively large heterozygous deletions [15]. The second mutation in these families may also have been located within the gene regulatory elements or unknown exons including alternative splicing areas. Although rare, a single *EYS* mutation in combination with another mutation on a second gene could also explain this phenotype [3].

The c.4957_4958insA and c.8868C>A mutations were not detected in Japanese patients with adRP or with LCA. Abd El-Aziz et al. reported that *EYS* gene mutation screening did not reveal any pathogenic mutations in 95 British and Chinese adRP patients [3]. Bandah-Rosenfeld et al. reported that no mutation was found in 2 Oriental Jewish and Israeli Muslim LCA patients who had a large homozygous region harboring the *EYS* gene [12]. Although further analysis of all *EYS* gene exons is required, *EYS* gene mutations may not be detected in Japanese patients with adRP and LCA. The c.4957_4958insA and c.8868C>A mutations were also detected in Korean patients with arRP and accounted for 6.3% (4/64 alleles) of the disease alleles. Similar to Japanese arRP results, the c.4957_4958insA mutation was more frequently detected than the c.8868C>A mutation. The fact that both c.4957_4958insA and c.8868C>A mutations were also detected in Korean patients suggests the possibility that the mutations occurred in an ancient common ancestor and spread throughout East Asia.

RP is a highly heterogeneous disease, with a reported prevalence rate of 1 in 4,000–8,000 people in Japan. Given the population of Japan, approximately a 100 million, the number of patients with RP can be estimated to be 12,500–25,000. The relative frequencies of RP inheritance patterns in Japanese patients were estimated as 25.2% for autosomal recessive, 16.9% for autosomal dominant, 1.6% for X-linked, and 56.3% for simplex, indicating that most Japanese RP patients represent arRP or isolated cases [17]. Autosomal recessive and simplex cases account over 80% of RP cases in Japan (approximately 10,000–20,000 people). Our results indicate that c.4957_4958insA and c.8868C>A mutations are possibly present in 1,600–3,200 Japanese patients with RP. These 2 novel mutations will be very useful for genetic diagnosis and counseling, and analysis of the mutated proteins may be helpful in the development of effective therapies for RP in Japan and Korea.

In conclusion, mutation screening of the *EYS* gene in 100 Japanese patients revealed 13 different pathogenic mutations, confirming that the mutation spectrum in Japanese patients differs from the previously reported spectrum in patients of non-Asian populations. Among these 13 mutations, 2 truncating mutations, c.4957_4958insA and c.8868C>A, were detected in at least one mutated allele in 16% of Japanese arRP patients and may be the most frequent mutations causing RP in the Japanese populations. Screening for c.4957_4958insA and c.8868C>A mutations in the *EYS* gene is, therefore, very effective for the genetic testing and counseling of RP patients in Japan. Further analysis is necessary to obtain a more precise mutation spectrum and to identify other frequent mutations in other East Asian populations.

## Supporting Information

Table S1 **PCR primer sequences for human** ***EYS*** **.**
(DOC)
