# Supplementary material for: Two Novel Mutations in the EYS Gene Are Possible Major Causes of Autosomal Recessive Retinitis Pigmentosa in the Japanese Population
Source: PLoS One. 2012 Feb 17;7(2):e31036. doi: 10.1371/journal.pone.0031036 (PMC3281914; doi:10.1371/journal.pone.0031036)
Supplement: Table S1 — PCR primer sequences for human EYS . (DOC) [file pone.0031036.s001.doc]

Supporting Information

Table S1. PCR primer sequences for human *EYS.*

*Primers used for mutation analysis in Japanese adRP and LCA patients, and Korean arRP patients.
